# Personality traits and loneliness in school-aged children with perceived hearing difficulties: results from a large-scale Norwegian study

**DOI:** 10.3389/fpsyg.2026.1693530

**Published:** 2026-08-31

**Authors:** Shahram Moradi, Lars Bauger, Christian Møller-Skau, Tore Norendal Braathen

**Affiliations:** 1 Department of Health, Social and Welfare Studies, Research Group for Disability and Inclusion, University of South-Eastern Norway, Porsgrunn, Norway; 2 Department of Health, Social and Welfare Studies, Research Group for Health Promotion in Settings, University of South-Eastern Norway, Tønsberg, Norway

**Keywords:** inclusive education, loneliness, perceived hearing difficulties, personality traits, pupil-reported data

## Abstract

**Objectives:**

This study aims to examine the associations between perceived hearing difficulties and both personality traits (extraversion, neuroticism, conscientiousness) and loneliness among school-aged children in Norway.

**Design:**

Data were drawn from an ongoing population-based survey conducted in primary schools in two Norwegian counties. The sample included 6,049 children between 10 and 13 years of age. Perceived hearing difficulties were self-reported via a single questionnaire item. Personality traits were measured through a set of self-report items. Mean scores were computed for each trait. Loneliness was assessed with a single-item measure on a 0–10 scale. Hierarchical regression analyses examined the associations between perceived hearing difficulties and each outcome. For personality traits, models were adjusted for gender and class grade; for loneliness, the analysis additionally adjusted for personality traits.

**Results:**

Children with perceived hearing difficulties had significantly lower conscientiousness and higher neuroticism scores than peers without perceived hearing difficulties. No significant association was found for extraversion. They also experienced higher levels of loneliness than peers without perceived hearing difficulties.

**Conclusion:**

Perceived hearing difficulties are associated with distinct personality profiles among school-aged children, characterized by higher emotional vulnerability and lower persistence (or goal-oriented behavior) and also higher levels of loneliness. These findings highlight the importance of supporting psychological and emotional well-being in inclusive education practices for children with hearing difficulties.

## Introduction

Globally, an estimated 34 million children live with disabling hearing loss (World Health Organization, 2021). Hearing difficulties in childhood can impair communication, learning, and social development, influencing not only language and cognitive development but also emotional and psychosocial adjustment (de Jong et al., 2024; Stevenson et al., 2015; Theunissen et al., 2014). These psychosocial challenges raise important questions about the broader developmental trajectories of children with hearing loss, particularly with regard to personality development. Personality traits, which reflect relatively stable patterns of thinking, feeling, and behaving, begin to emerge in early childhood and become more clearly established by middle childhood (Soto and Tackett, 2015; Tackett et al., 2012). Researchers have increasingly adapted the Five-Factor Model (or “Big Five”) to study personality in children. This framework, which emerged from decades of research, reduces thousands of trait descriptors to five core factors (extraversion, agreeableness, conscientiousness, neuroticism, and openness to experience) (Costa and McCrae, 1992; Goldberg, 1993). These traits are known to be associated with important outcomes in childhood, including academic success, social competence, and emotional well-being (Smrtnik-Vitulić and Zupančič, 2011; Soto and Tackett, 2015).

Although personality traits have been studied in relation to hearing loss among older adults (e.g., Cormier et al., 2024; Stephan et al., 2019), research on younger individuals remains limited. Most studies involving children and adolescents with hearing impairment have focused on emotional and behavioral difficulties or social functioning (de Jong et al., 2024; Theunissen et al., 2014) rather than on broad, stable personality traits. While emotional difficulties provide important insights into how children with hearing difficulties respond in specific contexts, they do not fully capture the broader patterns of thinking, feeling, and behaving that reflect personality traits. Even in childhood, personality traits reflect meaningful individual differences that extend beyond temporary emotional states or situational behaviors (Caspi et al., 2005; Soto and Tackett, 2015; Tackett et al., 2012). Of note, although personality remains more flexible and subject to developmental change during middle childhood and adolescence than in adulthood (Roberts and DelVecchio, 2000), this developmental period is also characterized by the emergence of early personality configurations (Soto and Tackett, 2015; Tackett et al., 2012; Caspi et al., 2005). Examining personality traits may therefore provide a more developmentally relevant understanding of how hearing difficulties relate to children’s emerging personality development and broader socio-emotional functioning. To best of our knowledge, only one study to date has investigated personality profiles in youth with hearing difficulties. Boerrigter et al. (2018) examined a cohort of adolescents with cochlear implants and found largely typical personality profiles, with only minor differences linked to speech perception and language comprehension. Boerrigter et al. (2018) focused exclusively on adolescents with profound hearing loss using cochlear implants. No studies have yet examined personality traits among younger children with hearing difficulties, leaving a critical gap in our understanding of how early hearing difficulties may relate to personality development.

In addition, one notable psychosocial consequence of childhood hearing impairment is loneliness. Children with hearing loss often face challenges in peer interactions and may feel socially isolated due to communication barriers [see the reviews by Patel et al. (2021) and Shukla et al. (2020)]. Reduced speech perception caused by hearing difficulties can impair social competence in interactions with peers who have normal hearing (Most et al., 2012), thereby increasing the risk of loneliness. Loneliness can shortly be described as a distressing feeling due to discrepancy between a persons desired and their actual social connections (Hawkley and Cacioppo, 2010) and has been linked to personality (Teppers et al., 2013). With the traits extraversion and neuroticism as the strongest predictors of loneliness (Buecker et al., 2020), where extraversion is negatively associated (particularly in younger age), whereas neuroticism shows a positive association with loneliness.

The present study examines the relationship between perceived hearing difficulties, personality traits, and loneliness in a large Norwegian sample of primary school children participating in the Ungdata Pluss study. Using data from children in two counties in South-Eastern Norway, the present study assessed perceived hearing difficulty with a self-report item from a translated version of the United Nations/Washington Group Child Functioning Module (Gulløy et al., 2023; Washington Group on Disability Statistics and UNICEF, 2020) and measured personality using child-adapted scales. The present study focused on three personality dimensions (extraversion, neuroticism, and conscientiousness) rather than all five domains of the Big Five model, primarily because the Ungdata Pluss survey was designed as a broad population-based study with limited space available for extensive personality assessment. Consequently, only selected personality dimensions considered most relevant to children’s socio-emotional functioning were available in the survey. These three dimensions have parallels in models of early childhood temperament, with sociability, negative emotionality, and persistence representing developmental precursors of extraversion, neuroticism, and conscientiousness, respectively (Soto and Tackett, 2015). Specifically, the authors focused on extraversion, characterized by sociability, energy, and a tendency to seek social interactions; neuroticism, capturing emotional vulnerability, including tendencies toward anxiety, sadness, and emotional instability; and conscientiousness, capturing persistence, self-discipline, and goal-directed behavior (Soto and John, 2017; Tackett et al., 2012). Additionally, the authors included a single-item measure of loneliness to capture an aspect of the children’s social well-being. Examining both personality traits and loneliness therefore allows for a broader understanding of the psychosocial functioning associated with perceived hearing difficulties in childhood. In other words, the present study not only explores whether children with perceived hearing difficulties differ in their personality traits, but also whether they experience greater feelings of social isolation in everyday life.

## Methods

### Study design and participants

This study used data from the Ungdata Pluss study, an ongoing longitudinal survey of children’s well-being, leisure activities, and development in Vestfold and Telemark counties in Norway (Ungdata, 2023). All primary school children in grades 5–7 (ages 10–13 years old) across these counties were invited to participate. The present analysis is based on the first data collection, conducted in spring 2023. A total of 6,049 children participated, corresponding to about 51% of the eligible population (Vardheim et al., 2023). Data were collected during regular school hours using a self-administered digital questionnaire. School administrators coordinated the survey within each participating school. Prior to data collection, written informed consent was obtained from parents or legal guardians of all children. Participation was voluntary, and children were informed that they could withdraw at any time without consequence.

### Ethical considerations

The study was approved by the Norwegian Agency for Shared Services in Education and Research (reference number: 237024). All identifying information was removed from the dataset to ensure participant anonymity.

## Measures

### Perceived hearing difficulties

Perceived hearing difficulties were assessed with a single self-report question translated and adapted from a version of the Washington Group Child Functioning Module (Gulløy et al., 2023; Washington Group on Disability Statistics and UNICEF, 2020): “Do you have trouble hearing?” Response options were: (1) No, never; (2) Yes, sometimes; (3) Yes, often; (4) All the time; and (5) Do not know. For the purposes of analysis, responses were dichotomized: children who answered “Yes, often” or “All the time” were classified as having perceived hearing difficulties, while those who answered “No, never” or “Yes, sometimes” were classified as not having perceived hearing difficulties. Responses of “Do not know” were treated as missing and excluded from analyses.

The WG/UNICEF CFM is an internationally developed survey module designed to identify functional difficulties among children in population-based surveys, and previous research has supported its construct validity and cross-national applicability in child disability research (Cappa et al., 2018; Loeb et al., 2018). The Norwegian version was translated following standard translation procedures to ensure linguistic and conceptual equivalence (Gulløy et al., 2023). However, the present study relied on a single self-report item assessing perceived hearing difficulties rather than objective audiometric assessment. As such, the measure captures subjective functional hearing difficulties in everyday situations and may be influenced by contextual and individual factors, including children’s cognitive capacities and background noise in school settings. In addition, the use of a single-item measure may not fully capture the multidimensional nature of hearing-related functioning, which can encompass listening effort, social participation, and environmental listening situations. Furthermore, single-item measures generally provide lower reliability than multi-item scales (Diamantopoulos et al., 2012; Wanous and Reichers, 1996). Nevertheless, single-item functional screeners are commonly used in large-scale population-based studies because of their feasibility and reduced respondent burden when clinical assessments are not possible (Madsen et al., 2025). These limitations are discussed further in the Limitations section. Of note, this item indexes perceived hearing difficulty in daily life and does not differentiate permanent hearing loss from temporary middle-ear conditions or potential auditory processing disorder. Consequently, some misclassification is possible, as perceived difficulties may also be influenced by psychological or attentional factors.

### Personality traits

As noted earlier, this study examined three personality trait domains: extraversion, neuroticism, and conscientiousness. Although Ungdata Pluss includes only two traits using regular personality measurement (BFI-2-S), We use the persistence dimension, from the EPOCH measure of well-being, as a proxy for the trait conscientiousness in this study. Each trait was measured using a set of child self-report items, with responses on a five-point Likert scale (1 = “Strongly disagree” to 5 = “Strongly agree”). Items were reverse-scored according to the published scoring keys of the respective instruments so that higher scores consistently indicate more of the trait.

*Extraversion* was measured with six items from the validated Norwegian version of the BFI-2-S developed by Føllesdal and Soto (2022). Example items include “I am often quiet” (reverse-scored) and “I am outgoing and sociable.” Cronbach’s alpha for this scale in the present Ungdata Pluss sample was: 0.59.

*Neuroticism* was measured with six items from BFI-2-S. Example items include “I am often worried,” “I can be depressed or down,” and “I get upset easily.” Some items were reverse-scored (e.g., “I am rarely anxious or scared,” reversed so that higher scores reflect higher neuroticism). Cronbach’s alpha for this scale in the present Ungdata Pluss sample was: 0.73.

Because our sample consisted of children, we conducted cognitive testing with children and made minor wording adjustments to ensure age-appropriate language understanding, while preserving the original meaning of the items. For example, the item “I tend to be quiet” (reverse-scored) was translated as “Jeg er stillferdig” in the Norwegian version of Føllesdal and Soto (2022), but we replaced “stillferdig” with “stille,” which is more commonly understood by children. Similarly, for neuroticism, we simplified phrases such as “Har en tendens til å være deprimert og nedfor” to “Jeg kan være deprimert og nedfor.” These changes were purely linguistic and did not alter the underlying constructs.

*Conscientiousness* was measured using four items from the EPOCH Measure of Adolescent Well-Being (Kern et al., 2016), which focuses on goal-directed persistence. Example items include “I finish everything I start” and “I work very hard,” reflecting the child’s perseverance and diligence, and closely aligned with the trait conscientiousness. Cronbach’s alpha for this scale in the present Ungdata Pluss sample was: 0.78.

### Loneliness

Loneliness was measured with a single item asking the children to rate how often they felt lonely. Each child was asked: “Think about how you have felt in the last 7 days. To what degree have you felt lonely?.” Responses were given on an 11-point scale ranging from 0 (“not at all”) to 10 (“very often”). Higher scores indicate more frequent feelings of loneliness in the past week.

### Statistical analysis

All analyses were conducted using IBM SPSS Statistics Version 29. First, we computed descriptive statistics (means, standard deviations, frequencies) for all key variables. We then ran four separate linear regression models to examine the associations between perceived hearing difficulties and each outcome: extraversion, neuroticism, conscientiousness, and loneliness.

Prior to conducting the regression analyses, assumptions for linear regression were evaluated for the fully adjusted models. Multicollinearity among predictors was examined using variance inflation factors (VIF), and no evidence of problematic multicollinearity was observed (all VIF values were low and close to 1.0). Histograms and normal probability plots of standardized residuals indicated that residuals were approximately normally distributed, with only minor deviations observed in some models. Given the large sample size and cross-sectional nature of the data, the regression models were considered sufficiently robust to minor assumption deviations. Overall, the assumptions for linear regression were judged to be adequately met. Categorical variables were entered into the regression analyses using binary coding procedures. Gender was coded as a dichotomous variable (boys = 1, girls = 2), and perceived hearing difficulties were coded as a binary predictor (1 = no perceived hearing difficulties [never/sometimes], 2 = with perceived hearing difficulties [often/all the time]). Grade level was coded ordinally to reflect increasing school grade (1 = grade 5, 2 = grade 6, 3 = grade 7).

Each analysis followed a two-step modeling approach: In Model 1, perceived hearing difficulties were entered as the sole predictor to assess unadjusted associations with each outcome variable. In Model 2, we added control variables: gender and class grade for the personality trait outcomes, and gender, class grade, and the three personality traits (extraversion, neuroticism, conscientiousness) for the loneliness outcome. It should be noted that this study is part of an ongoing population survey. All pupils in grades 5–7 in Vestfold and Telemark counties were invited to participate in the spring of 2023; therefore, no *a priori* power calculation was conducted. All regression estimates are presented with 95% confidence intervals.

Missing data were handled using listwise deletion. A significance level of *p* < 0.05 was used for all statistical tests. The number of missing individuals for the variables in this study are as follows: perceived hearing function, *n* = 569; extraversion, *n* = 231; neuroticism, *n* = 244; conscientiousness, *n* = 513; loneliness, *n* = 243; gender, *n* = 22; class grade, *n* = 12.

## Results

Descriptive statistics for the sample are presented in Table 1. Perceived hearing difficulties were reported slightly more often by girls than boys, with the highest proportion found among girls in grade 7. In terms of personality traits, girls reported higher average scores on neuroticism than boys. Boys, on the other hand, reported slightly higher levels of extraversion and conscientiousness. Across grade levels, a small decline was observed in extraversion and conscientiousness, while neuroticism increased slightly, particularly among girls. Girls also reported higher levels of loneliness than boys, with the highest loneliness scores observed among girls in grade 7.

**Table 1:** Sample characteristics (*N* = 6,049).

	Class grade 5	Class grade 6	Class grade 7	Total
Girls	(*n* = 1,028)	(*n* = 979)	(*n* = 965)	(*n* = 2,972)
Perceived hearing difficulties (%)	(2.2)	1.1	4.0	2.4
Extraversion (SD)	3.65 (0.64)	3.59 (0.57)	3.45 (0.70)	3.56 (0.67)
Neuroticism (SD)	2.65 (0.67)	2.72 (0.66)	2.81 (0.71)	2.73 (0.68)
Conscientiousness (SD)	3.62 (0.73)	3.51 (0.72)	3.35 (0.78)	3.50 (0.75)
Loneliness	3.04 (2.65)	2.95 (2.41)	3.43 (2.69)	3.14 (2.60)
Boys	(*n* = 965)	(*n* = 1,067)	(*n* = 1,011)	(*n* = 3,043)
Perceived hearing difficulties (%)	1.1	2.0	2.0	1.7
Extraversion (SD)	3.67 (0.66)	3.68 (0.64)	3.63 (0.68)	3.66 (0.66)
Neuroticism (SD)	2.39 (0.65)	2.39 (0.64)	2.39 (0.62)	2.39 (0.64)
Conscientiousness (SD)	3.64 (0.74)	3.58 (0.74)	3.57 (0.75)	3.59 (0.74)
Loneliness (SD)	2.40 (2.37)	2.24 (2.01)	2.52 (2.29)	2.38 (2.24)

SD, standard deviation.

Multiple hierarchical regression analyses were conducted to examine the associations between perceived hearing difficulties and personality traits, controlling for gender and grade level. The results are presented in Table 2.

**Table 2:** Hierarchical linear regressions predicting personality traits of extraversion, neuroticism, conscientiousness, and loneliness.

	Extraversion *B*	Neuroticism *B*	Conscientiousness *B*	Loneliness *B*
Step 1				
Constant	3.76 (3.63–3.89)	2.21 (2.08–2.35)	3.87 (3.72–4.02)	1.10 (0.60–1.59)
Perceived hearing difficulties	**-0.13 (−0.26 to −0.01)**	**0.32 (0.19–0.45)**	**−0.30 (−0.45 to −0.16)**	**1.60 (1.12–2.09)**
*R*^2^ (Step 1)	0.001	0.004	0.003	0.008
Step 2				
Constant	3.99 (3.85–4.14)	1.69 (1.55–1.83)	4.14 (3.98–4.31)	−0.13 (−0.86 to 0.59)
Perceived hearing difficulties	−0.11 (−0.24–0.01)	**0.28 (0.16–0.41)**	**−0.28 (−0.42 to −0.13)**	**0.95 (0.52–1.37)**
Gender	**−0.09 (−0.13 to −0.05)**	**0.33 (0.29–0.36)**	**−0.10 (−0.14 to −0.06)**	**0.21 (0.09–0.34)**
Class grade	**-0.06 (−0.08 to −0.04)**	**0.04 (0.02–0.06)**	**−0.08 (−0.10 to −0.05)**	0.07 (−0.01–0.14)
Extraversion	–	**–**	**–**	−**0.49 (−0.59 to −0.40)**
Neuroticism	–	**–**	**–**	**1.44 (1.35–1.54)**
Conscientiousness	–	**–**	**–**	**−0.12 (−0.21 to −0.04)**
*R*^2^ (Step 2)	0.01	0.064	0.014	0.239

Unstandardized regression coefficients (*B*) with 95% confidence intervals in parentheses. Bold text indicates statistical significance at *p* < 0.05. Gender coded as 1 = boy, 2 = girl. Perceived hearing difficulty coded as 1 = without hearing difficulties, 2 = with hearing difficulties. Class grade was treated ordinally to reflect increasing school grade.

### Personality traits

#### Extraversion

A hierarchical multiple linear regression was conducted to examine the association between perceived hearing difficulties and extraversion. In Model 1, perceived hearing difficulties were entered as the sole predictor. The model was statistically significant, *F*(1, 5,341) = 4.28, *p* = 0.039, although it accounted for a very small proportion of the variance in extraversion (*R*^2^ = 0.001). Children with perceived hearing difficulties scored significantly lower on extraversion than those without [*B* = −0.134, SE = 0.065, *p* = 0.039, 95% CI (−0.261 to −0.007)].

In Model 2, gender and class grade were added as control variables. The full model remained statistically significant, *F*(3, 5,339) = 18.64, *p* < 0.001, with *R*^2^ of 0.010. In this adjusted model, there was no significant association between perceived hearing difficulties and extraversion [*B* = −0.113, SE = 0.065, *p* = 0.079, 95% CI (−0.240 to 0.013)]. However, both gender and class grade emerged as significant predictors: girls reported lower extraversion than boys [*B* = −0.089, SE = 0.018, *p* < 0.001, 95% CI (−0.125 to −0.054)], and extraversion scores decreased slightly with increasing grade level [*B* = −0.060, SE = 0.011, *p* < 0.001, 95% CI (−0.082 to −0.038)].

#### Neuroticism

A hierarchical multiple linear regression was conducted to examine the association between perceived hearing difficulties and neuroticism. In Model 1, perceived hearing difficulties were entered as the sole predictor. The model was statistically significant, *F*(1, 5,331) = 23.88, *p* < 0.001, explaining 0.4% of the variance in neuroticism (*R*^2^ = 0.004). Children with perceived hearing difficulties reported significantly higher levels of neuroticism than those without perceived hearing difficulties [*B* = 0.320, SE = 0.065, *p* < 0.001, 95% CI (0.192–0.448)].

In Model 2, gender and class grade were added as control variables. The full model was statistically significant, *F*(3, 5,329) = 122.34, *p* < 0.001, with an increased *R*^2^ of 0.064. A significant association was observed between perceived hearing difficulties and neuroticism [*B* = 0.282, SE = 0.064, *p* < 0.001, 95% CI (0.158–0.407)]. Girls scored higher than boys [*B* = 0.328, SE = 0.018, *p* < 0.001, 95% CI (0.293–0.363)], and children in higher grades reported slightly more neuroticism [*B* = 0.037, SE = 0.011, *p* < 0.001, 95% CI (0.015–0.059)].

#### Conscientiousness

A hierarchical multiple linear regression was conducted to examine the association between perceived hearing difficulties and conscientiousness. In Model 1, perceived hearing difficulties were entered as the sole predictor. The model was statistically significant, *F*(1, 5,090) = 16.56, *p* < 0.001, although it explained a small proportion of the variance in conscientiousness (*R*^2^ = 0.003). Children with perceived hearing difficulties scored significantly lower on conscientiousness than those without perceived hearing difficulties [*B* = −0.304, SE = 0.075, *p* < 0.001, 95% CI (−0.451 to −0.158)].

In Model 2, gender and class grade were added as control variables. The full model was statistically significant, *F*(3, 5,088) = 24.52, *p* < 0.001, and explained 1.4% of the variance (*R*^2^ = 0.014). A significant association was observed between perceived hearing difficulties and conscientiousness [*B* = −0.278, SE = 0.074, *p* < 0.001, 95% CI (−0.424 to −0.132)]. Girls scored lower than boys [*B* = −0.096, SE = 0.021, *p* < 0.001, 95% CI (−0.137 to −0.055)], and children in higher grades scored slightly lower [*B* = −0.078, SE = 0.013, *p* < 0.001, 95% CI (−0.103 to −0.053)].

#### Loneliness

A hierarchical multiple linear regression was conducted to examine the association between perceived hearing difficulties and loneliness. In Model 1, perceived hearing difficulties were entered as the sole predictor. The model was statistically significant, *F*(1, 4,982) = 42.32, *p* < 0.001, and explained 0.8% of the variance in loneliness (*R*^2^ = 0.008). Children with perceived hearing difficulties reported significantly higher levels of loneliness than those without [*B* = 1.603, SE = 0.246, *p* < 0.001, 95% CI (1.120–2.087)].

In Model 2, gender, class grade, and the three personality traits (extraversion, conscientiousness, and neuroticism) were added as control variables. The full model was statistically significant, *F*(6, 4,977) = 260.95, *p* < 0.001, and explained 23.9% of the variance in loneliness. After controlling for these variables, an association was observed between perceived hearing difficulties and loneliness [*B* = 0.946, SE = 0.217, *p* < 0.001, 95% CI (0.521–1.372)]. Girls reported significantly higher loneliness than boys [*B* = 0.214, SE = 0.062, *p* < 0.001, 95% CI (0.092–0.336)], while class grade showed a non-significant trend [*B* = 0.068, SE = 0.038, *p* = 0.070, 95% CI (−0.006 to 0.142)].

Among the personality traits, extraversion was negatively associated with loneliness [*B* = −0.493, SE = 0.048, *p* < 0.001, 95% CI (−0.588 to −0.398)], neuroticism was positively associated [*B* = 1.444, SE = 0.049, *p* < 0.001, 95% CI (1.348–1.540)], and conscientiousness was negatively associated [*B* = −0.124, SE = 0.043, *p* = 0.004, 95% CI (−0.208 to −0.040)].

## Discussion

The findings indicate that children who experience perceived hearing difficulties experience more loneliness and exhibit a distinctive pattern of personality traits, compared to their peers without perceived hearing difficulties. The findings are in line with broader evidence that people with hearing difficulties tend to experience more loneliness compared to their peers without hearing difficulties (Patel et al., 2021; Shukla et al., 2020). The elevated levels of neuroticism observed among children with perceived hearing difficulties align with previous research indicating that such children are at increased risk for emotional and behavioral challenges, including anxiety and social isolation (de Jong et al., 2024; Olsson et al., 2018; Stevenson et al., 2015). We reason that the lower conscientiousness reported among children with perceived hearing difficulties may reflect reduced task persistence, self-regulation, or difficulties with maintaining focus and completing schoolwork. Further, we argue that the cognitive and communicative challenges associated with hearing difficulties (such as increased listening effort, frequent misunderstandings, and difficulties following instructions in daily activities) may impede the development of conscientious behaviors in school-aged children.

The findings both align with and differ from those of Boerrigter et al. (2018), who reported largely comparable personality profiles in adolescents using cochlear implants and normal-hearing peers, with only minor differences linked to speech perception and language comprehension. In contrast, our results, based on a broader group of school-aged children with perceived hearing difficulties, presumably with varying degrees of hearing loss, and not necessarily using cochlear implants, showed significant differences in neuroticism and conscientiousness. These discrepancies may reflect differences in age, hearing loss severity, and assistive technology use, and educational context. Adolescents with cochlear implants in Boerrigter et al.’s (2018) study may have benefited from early targeted auditory or other rehabilitation strategies, whereas younger children in this sample may not have had such support, potentially influencing the early formation of personality traits.

The lack of a significant association between perceived hearing difficulties and extraversion suggests that, despite communication barriers, children with perceived hearing difficulties did not report lower sociability or outgoingness compared to their peers. One possible explanation is that Norway’s inclusive education policies (emphasizing inclusion, equity, and social integration) may help protect children with special needs from social isolation (European Agency for Special Needs and Inclusive Education, n.d.; Uthus and Qvortrup, 2025). However, the reasons for this non-association remain unclear and call for future research. At the same time, the persistence of significant association in loneliness, neuroticism, and conscientiousness—despite the inclusive context—indicates that solely educational inclusion alone may not be sufficient to compensate for all aspects of positive personality development or to reduce the feelings of loneliness.

We argue that beyond inclusive education, targeted psychosocial interventions may be necessary to reduce loneliness, enhance emotional resilience, and support the development of self-discipline and goal-directed behavior in children with hearing difficulties. For example, in line with interventions known to increase conscientiousness (Javaras et al., 2019) schools can strengthen these children’s organizational skills and task persistence. Similarly, emotional resilience can be enhanced through early intervention programs, counseling services, and classroom practices designed to reduce anxiety and feeling of loneliness [see the reviews by Fenwick-Smith et al. (2018) and Twum-Antwi et al. (2020)]. By promoting conscientious habits alongside emotional support, educators can create inclusive settings that promote psycho-social well-being for children with hearing difficulties.

One apparent contradiction in the findings is that, while no significant association was found between the two groups on extraversion, children with perceived hearing difficulties reported significantly higher levels of loneliness. It is important to consider measurement-related factors that may help explain this discrepancy. The six extraversion items used in this study primarily assessed general social confidence, energy, and behavioral engagement (e.g., “I am often quiet,” “I have a lot of energy,” “I am outgoing and social”), rather than the depth and quality of social–emotional relationships with peers. We argue that children with hearing difficulties may report similar levels of extraversion as their peers with normal hearing, because of their continued social motivation. However, their social efforts may not lead to fulfilling or reciprocal peer connections, especially when communication barriers are present. In contrast, the single-item measure of loneliness directly captured the subjective social and emotional experience of feeling alone or social disconnection (“To what degree have you felt lonely in the last 7 days?”). In fact, prior research emphasizes that loneliness is not merely about having social contact, but about the depth, reciprocity, and fulfillment of those interactions (Qualter et al., 2015), as well as an alignment between desired and actual social relationships (Hawkley and Cacioppo, 2010). In their theoretical framework, Qualter et al. (2015) introduce the Reaffiliation Motive (RAM), which suggests that loneliness triggers a desire to reconnect with others. However, if attempts at reconnection are met with rejection or fear of negative evaluation (e.g., due to communication barriers, as may occur for children with hearing difficulties), this reaffiliation process can break down, leading to social withdrawal. In this context, children may still exhibit high levels of extraversion, reflecting a strong desire for social engagement, while simultaneously feeling lonely due to unsuccessful or unsatisfying peer interactions.

Norway’s education system is grounded in the Nordic model of inclusion and is closely aligned with the principles of the UN Convention on the Rights of Persons with Disabilities (CRPD; United Nations, 2006). National policies emphasize equal participation, adapted education, and universal design in schools, supported by collaboration between teachers, special educators, and welfare services. These structures may partly buffer the social and emotional challenges associated with hearing difficulties. However, comparable inclusive frameworks are not equally developed in many other countries, and the generalizability of our findings should therefore be interpreted with caution. Future cross-national studies are needed to examine how different levels of educational inclusivity shape psychosocial outcomes for children with hearing difficulties.

Despite the strengths of the large sample, this study has several limitations. First, hearing status was based on a single self-report item from the WG/UNICEF CFM rather than audiometry. Thus, children with reported difficulty may include those with permanent hearing loss, temporary middle-ear pathology (e.g., otitis media), or auditory processing disorder, or sensory hypersensitivity such as hyperacusis (particularly among children with autism spectrum disorder). As a result, it was not possible to distinguish between permanent sensorineural loss and transient or perception-related difficulties. Future studies should include objective audiological assessments and clinical history to distinguish these conditions. In addition, although dichotomizing the hearing item simplified interpretation and allowed for group comparisons between children with and without perceived hearing difficulties, this approach may have led to a loss of information about variability in hearing experiences. Second, both hearing difficulties and loneliness were each assessed with a single self-reported item. Although single-item indicators are commonly used in large population surveys to reduce respondent burden (e.g., Madsen et al., 2025), they may provide less reliable and valid assessments than standardized multi-item instruments. In particular, the single-item measure of loneliness may not fully capture the multidimensional nature of loneliness among children. Future studies should consider using validated multi-item scales such as the Children’s Loneliness and Social Dissatisfaction Scale (Asher et al., 1984) or the Loneliness and Aloneness Scale for Children and Adolescents (LACA) (Goossens and Maes, 2017), which have demonstrated strong psychometric properties and are well suited for children and adolescents. Third, the internal consistency of the six-item Extraversion scale was modest (Cronbach’s *α* = 0.59), falling below the conventional 0.70 threshold and suggesting that random measurement error may have reduced the strengths of associations involving this trait. Fourth, cross-sectional design limits causal inference. The authors cannot determine whether perceived hearing difficulties lead to the observed personality differences and loneliness. Because perceived hearing difficulties and psychosocial outcomes were assessed simultaneously using self-report measures, inverse or bidirectional relationships cannot be ruled out. It is possible that emotional vulnerability or attentional difficulties increase children’s perception of hearing-related problems rather than result from them, highlighting the need for further research. The findings should therefore be interpreted as relational associations rather than evidence of causal effects. Future longitudinal research incorporating objective hearing assessments may help to clarify directionality. Fifth, the authors examined only three personality traits (neuroticism, extraversion, conscientiousness); including agreeableness and openness to experience in future research might reveal additional patterns. Sixth, although the effect sizes for neuroticism and conscientiousness were statistically significant, the low *R*^2^ values indicate that perceived hearing difficulties and the included covariates account for only a small part of the variation in these personality traits. Seventh, the authors did not collect information on the use of cochlear implants, other specific hearing devices, or sign language for communication. Such data could have provided valuable information for understanding variability in psychosocial outcomes among children with perceived hearing difficulties. Eighth, all variables were based on pupil self-report collected in the same survey session. This may have introduced shared method variance, which can inflate associations between constructs. Future research should integrate self-reports with teacher or parent assessments to enhance measurement validity. Ninth, one important point to note is that the sample in this study consisted of children aged 10–13 years, a developmental period during which emotional, motor, language, and cognitive capacities continue to develop, leading to personality traits relatively malleable and still undergoing maturation (Caspi et al., 2005). Consequently, the observed patterns in personality traits in our study should not be interpreted as stable characteristics. In combination with the cross-sectional design, this developmental context further limits causal interpretations regarding personality development. Finally, generalizability may be limited by the Norwegian context, with its strong emphasis on inclusive education for all children. Children in other educational environments might experience different challenges and supports, potentially leading to different associations between perceived hearing difficulties, personality traits, and loneliness.

In conclusion, perceived hearing difficulties in school-aged children are associated with meaningful differences in children’s social–emotional well-being. Early identification of these difficulties, combined with appropriate auditory accommodations and supportive interventions targeting loneliness, psycho-social well-being, and planning and persistence skills, can foster inclusion for children with hearing difficulties. Teachers and school counselors can do this in practice by fostering peer inclusion and a supportive classroom climate, using flexible communication strategies (e.g., written or visual instructions), and watching for subtle signs of loneliness or withdrawal so that psychosocial support is offered early.

## Data Availability

The datasets presented in this article are not readily available because only authorized researchers affiliated with a Norwegian university can obtain data. Requests to access the datasets should be directed to https://www.ungdata.no/pluss/.
